# Concerns over cardiovascular manifestations associated with monkeypox immunization: a literature review

**DOI:** 10.1097/MS9.0000000000000861

**Published:** 2023-05-19

**Authors:** Abhigan Babu Shrestha, Aashna Mehta, Muhammad Jawad Zahid, Katherine Candelario, Sajina Shrestha, Pashupati Pokharel

**Affiliations:** a M Abdur Rahim Medical College, Dinajpur, Bangladesh; b University of Debrecen-Faculty of Medicine, Debrecen, Hungary; c Department of General Surgery, Hayatabad Medical Complex, Peshawar, Pakistan; d Clinical Outcome Research Group, Division of Cardiac Surgery, Yale University, New Haven, CT; e KIST Medical College, Patan, Nepal; f Maharajgunj Medical Campus, Institute of Medicine, Kathmandu, Nepal

**Keywords:** adverse events, cardiac manifestations, immunization, Monkeypox

## Abstract

Following the recent surge in monkeypox cases worldwide, vaccines have gained popularity due to their protective role against the disease in the absence of specific pharmacological therapies for the disease. This article explores the possible challenges associated with the treatment and prevention of monkeypox with special emphasis on cardiovascular concerns relating to vaccines, warranting further investigation and research.

**Methods:**

Multiple electronic databases from inception till October 2022 were searched to find articles reporting the cardiovascular adverse events associated with monkeypox immunization.

**Results:**

Smallpox vaccines ACAM2000, JYNNEOS, and modified vaccinia Ankara have been used for monkeypox. ACAM2000 has been reported to have major cardiovascular adverse events such as myocarditis, dilated cardiomyopathy, and heart failure. Whereas JYNNEOS and modified vaccinia Ankara are associated with minor cardiovascular adverse events such as tachycardia, palpitation, electrocardiogram changes such as T wave inversion, and ST elevation.

**Conclusions:**

Despite having cardiovascular issues with the existing vaccines, clinicians and public health experts should measure the risk benefit aspect of smallpox vaccines to decide whether to go for mass immunization or not. Based on the lessons learned from the COVID-19 pandemic, immunization will be a better strategy to halt the monkeypox spread throughout the globe. However, further research is needed to determine the exact incidence and susceptibility to develop cardiovascular complications among monkeypox immunized individuals.

## Introduction

Highlights

- Recent surge in monkeypox cases, has raised concerns over the safety of pox vaccines globally.
- Pericarditis, myocarditis, and dilated cardiomyopathy are the major cardiovascular concerns in a person immunized for monkeypox.
- In a monkeypox vaccinated patient with chest pain, cardiac panel workup is recommended.

Human monkeypox virus (MPX) is an enveloped, double-stranded DNA virus of the *Orthopoxvirus* genus of the Poxviridae family^1^. Following the eradication of smallpox, MPX has become one of the most prevalent orthopox viruses^2^. Vast wild animal reservoirs, natural history, and pathogenesis of MPX in both animals and people remain unknown, necessitating definition through ecological and epidemiological studies^3^.

The first outbreak occurred in monkeys confined at a research facility in Copenhagen, Denmark, 1954^4^. As of 12 January 2023, more than 84 639 monkeypox cases have been reported from 110 countries along with 80 monkeypox related deaths worldwide^5^. United States of America (*n*=29 761), Brazil (*n*=10 599), and Spain (*n*=7505), have reported the highest number of cases, respectively. Incidences in the US, Israel, and Singapore have been associated with overseas travel or import of infected animals from endemic regions of Africa^6^. Interesting to note is the present MPX outbreak, that is disproportionately affecting non-endemic countries of Europe and the Americas. Human-to-human contact and transmission via respiratory droplets are the proposed hypothesis for the high number of monkeypox cases in these countries. Most of these cases have been linked to sexual transmission, with more noticeably affecting homosexual men in these regions^7^.

While treatment remains mostly supportive, several vaccines are available and gaining popularity due to the recent surge in cases worldwide. Currently, three smallpox vaccines, ACAM2000, JYNNEOS, and modified vaccinia Ankara, have been used by different authorities for monkeypox. These vaccines were initially developed for smallpox but are currently proven to be effective for monkeypox. These vaccines have a variable degree of cardiovascular issues to be considered. So, our review highlights the recent surge in MPX cases, the role of vaccines, and concerns related to cardiovascular manifestations associated with the MPX vaccine.

## Methods

This literature review is an evidence-based rapid review, which is similar to our previous article on monkeypox vaccines with neurological manifestations^8^. The purpose of this review is the first to scrutinize and retrieve all available articles on Monkeypox vaccines and potential cardiovascular manifestations. Electronic databases (PubMed, Embase, and Google Scholar) and Google search engine with relevant search terms: “Monkeypox Vaccine”, “cardiovascular”, “pericarditis”, “myocarditis”, and “small pox vaccine” were searched from inception till October 2022. Articles were compiled in Google sheet and all authors helped in data extraction and manuscript writing.

## Results

### Clinical presentation and diagnosis of monkeypox

The severity of MPX infection can range from moderate to lethal^9^. The incubation period normally falls between 5 and 21 days with patients generally presenting with fever, headache, muscle aches, and lethargy followed by centrifugal development of deep, well-circumscribed macular-papular, vesicular, pustular, eventually evolving into crusty scab lesions^9,10^. Unlike smallpox, lymphadenopathy has no specific pattern in relation to the occurrence of other symptoms and lesions typically erupt and recede simultaneously in each anatomic area^11^. Diagnosis is based on clinical symptoms which can be confirmed by laboratory tests such as polymerase chain reaction. The treatment for MPX remains mainly supportive in the absence of specific pharmacological drugs, while immunization in the form of vaccines is available and plays an active role in prevention of the disease. In fact, the frequency and severity of clinical signs and symptoms can be significantly reduced incase patients who have residual immunity from previous vaccination^12^.

Till date, much information has not been documented whether MPX has affinity to show cardiac tropism. A recent study of MPX showed two cases being hospitalized due to myocarditis and a case of a 31-year-old man confirmed with MPX having myocarditis^13,14^. Although the patient did not suffer much complications probably due to the benign nature of the disease. However, this has aroused the question if MPX really has an association with the cardiovascular system.

### History of cardiovascular events observed after vaccination

In regard to the cardiovascular complications caused by ACAM2000 vaccination, myocarditis, pericarditis, arrhythmias, and dilated cardiomyopathy (DCM) are common findings that have been discovered over the past decade. According to a study in the United States, healthy adult primary vaccine recipients of ACAM2000 have been reported to have an incidence of myocarditis and/or pericarditis at a rate of around 5.7 per 1000, 95% CI: 1.9–13.3^15^.

In previous study among 37 901 vaccinated US civilians (with licensed NYCBOH strain vaccine) on 2003, 5.5 per 10 000 cases of myo/pericarditis were observed. The median time of symptoms observed was 11 days and severities were mild, with no fatalities. Nevertheless, 43% were hospitalized. Furthermore, after 3 months, cases of dilated cardiomyopathy were recorded^2^. Along this line, few studies have delineated the incidence of overall myocarditis incidence and “vaccinia myocarditis”. The study of Karjalainen and Heikkila reported the overall incidence of myocarditis of any aetiology among military personnel to be 1.3 cases per 10 000 persons^1^. Contrary to this, the study of Halsell and colleagues reported the overall incidence of myo/pericarditis for any 30-day period among US military personnel conscripts to be 0.21 cases per 10 000 persons^3^. The incidence among Finnish military was recorded to be 1 per 10 000 persons^4^. Karjalainen and Heikkila observed the incidence of dilated cardiomyopathy among Finnish military personnel conscripts to be 0.02 per 1000 person-years^1^. Similarly, other studies have reported similar findings of the association of cardiovascular outcomes with smallpox vaccine^16–18^.

However, these studies have pointed out the presence of subclinical myo/pericarditis to be a more common phenomenon than clinical. A study reported over two-fold increase in cardiac specific troponin T (cTnT) (>99th percentile) from baseline (pre-smallpox vaccine) in subclinical myocarditis. This rate was 60 times higher than the background incidence rate of overt myocardial clinical cases^16^. Similarly, a study reported among US military personnel a myocarditis occurrence rate of 1 per 12819 primary vaccines and showed that a greater incidence was observed on the first vaccination than per vaccinated people. This could mean that many more immune reactions develop during the first vaccination and requires caution^17^. Table 1 summarizes the key cardiovascular events occurring after vaccination.

**Table 1:** Cardiovascular manifestations of monkeypox vaccines.

S.N.	Vaccine	Cardiovascular manifestation
1	ACAM2000 (major cardiac manifestation)	Myocarditis, dilated cardiomyopathy, heart failure
2	JYNNEOS and Modified Vaccinia Ankara (MVA) (minimum cardiac manifestation)	Tachycardia, palpitations, abnormal ECG findings: T wave inversion or ST elevation

ECG, electrocardiogram.

### Challenges associated with treatment and prevention of MPX

Due to the relative lack of specific pharmacological therapy, MPX is mainly managed with supportive treatment. As necessary, secondary bacterial superinfection should be treated. Recently, the European Medicines Agency (EMA) has authorized tecovirimat, an antiviral drug designed for smallpox, for monkeypox treatment^12^. Cidofovir and vaccinia immunoglobulin intravenous are also currently being utilized as conjuncts in serious cases^17^.

According to estimates, the smallpox vaccine offers about 85% protection against monkeypox^12^. The US Strategic National Stockpile (SNS) now contains three smallpox vaccines: JYNNEOSTM and ACAM2000 are licensed for smallpox, while the Aventis Pasteur Smallpox Vaccine (APSV) might be used for smallpox under an investigational new drug process. Both JYNNEOSTM and ACAM2000 are now suggested for persons 18 years of age and older who have been shown to be at high risk for contracting smallpox or MPX disease^18^.

According to CDC, a live replicating Vaccinia virus preparation called ACAM2000 is injected into the skin by pricking the skin surface, whereas JYNNEOSTM is a non-replicating live virus vaccine administered subcutaneously. ACAM2000 only requires one dose; however, JYNEOSTM requires two doses separated by 4 weeks. People who get the ACAM2000 vaccine must take precautions to stop the vaccine virus from spreading and are deemed immunized after 28 days. JYNNEOSTM recipients are not considered immunized until two weeks after receiving the second dose^19^.

Moreover, ACAM2000 can cause progressive vaccinia and eczema vaccinatum especially in immunocompromised individuals due to unchecked viral replication. Foetal vaccinia can also be caused by vertical transmission, which could be lethal to the foetus or infant^15^. In addition, myopericarditis and post-vaccination encephalitis are other severe side effects more commonly reported with ACAM2000® than with JYNNEOSTM^18^.

Severe complications and sequelae are more commonly observed among unvaccinated (74%) compared to vaccinated patients (39.5%)^13^. Late during the disease, patients frequently develop respiratory discomfort or pneumonia in case of a superimposed lung infection. By the second week of the disease, vomiting or diarrhoea may start, which can significantly worsen dehydration. Patients may rarely develop encephalitis. More than 4500 cases of septicaemia have been reported in Monkeypox infected individuals, with case fatality rates ranging from 0 to 11% in unvaccinated individuals^9,13^.

### Cardiovascular concerns associated with the MPX vaccines

The ACAM2000 has shown a number of reported adverse effects. The skin, eyes, heart, and nervous system can be affected. Urticaria, rashes, eczema vaccinatum, generalized vaccinia, and progressive vaccinia are common skin findings^20^. Considering ocular effects, ocular vaccinia is also a common finding. Encephalitis that occurs post vaccination, otherwise known as post-vaccinal encephalitis, is rare but fatal. Complications vary by age, but they tend to occur more commonly in those who are less than 12 months old^20^.

Myocarditis includes inflammation of the muscular layer of the heart, while pericarditis is the inflammation of the fibrous sac around the heart^21^. Myocarditis does not present with pathognomic features during its subclinical state, which means the disease can present late and with complications such as reduced heart function. In addition, myocarditis may even present with initial unexplained DCM and Heart failure. DCM, which is a weakening of heart muscle with dilation and enlargement of heart chambers, is another serious condition. When either left ventricular dysfunction occurs, or primary cardiomyopathy ensues, cardiac remodelling occurs as an attempt to maintain normal function. Through fibrosis and hypertrophy of the tissue, systolic dysfunction may result. Both of these conditions can cause palpitations, shortness of breath, fever, and chest pain, among other symptoms^21^. These cardiovascular sequelae can be evaluated with an electrocardiogram, echocardiogram, MRI, cardiac markers, and by testing the tissue pathologically, which is rarely performed. These inflammatory conditions can lead to many further complications, including fulminant congestive heart failure^21^.

**Figure 1:**
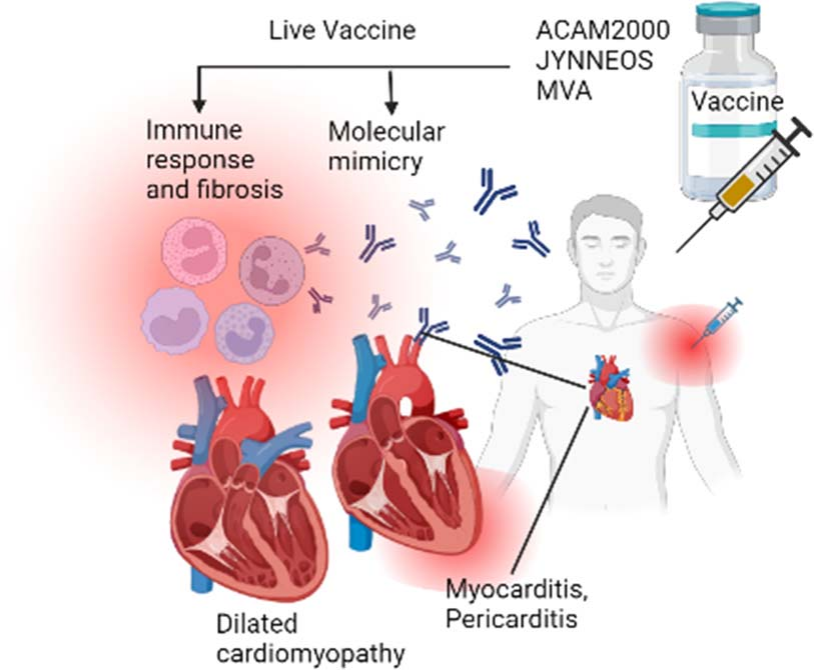
Pathophysiology of cardiac events associated with Monkeypox immunization.

The JYNNEOS vaccine and the modified vaccinia Ankara vaccine have shown to present with minor cardiovascular side effects including tachycardia, palpitations, abnormal electrocardiogram, T wave inversion or ST elevation. Other minor reactions include redness, swelling, pain, and itching at the injection site. Fever might also occur and patients may rarely develop severe allergic reactions^22^. Despite two possible speculations, including if it occurs as a direct viral cytopathogenic effect or an immune-mediated disease reaction, the exact pathomechanism by which cardiovascular pathology develops in response to MPX vaccine remains unclear^17^. In 2022 Sharff and colleagues analyzed the cardiovascular adverse events in 2126 patients who were immunized with the JYNNEOS vaccine. A total of 10 patients presented with complaints of chest pain or palpitation, and were reported to have cardiovascular adverse events^23^. Figure 1 shows the pathophysiology of cardiac events due to Monkeypox immunization.

## Recommendations and conclusions

Smallpox vaccine has associated risk to cardiovascular outcomes due to myo/pericarditis and DCM. Hence, for health policy makers, caution should be taken before considering mass vaccination for MPX campaigns given the clinical morbidity and costs associated with a case of myopericarditis. Risk of myo/pericarditis should be weighed carefully against the risk of exposure to MPX. For a physician encountering MPX vaccinated patients with chest pain, a detailed history and physical examination, electrocardiogram, and cardiac biomarkers in the initial step must be done along with prompt reporting to the Vaccine Adverse Events Reporting System. All patients should be monitored up to 21 days after infection, unless complications occur. Echocardiogram, magnetic resonance imaging, nuclear imaging, cardiac catheterization, and even cardiac biopsy may be warranted in further evaluation. Treatment should be carried out with non-steroidal anti-inflammatory agents, 4–6 weeks of limited exertion, and with the latest guideline in case of heart failure as required. The role of immunosuppressive therapy with steroids may be beneficial in myo/pericarditis associated with MPX vaccination compared to the conventional treatment for myocarditis of other types. Protocols as such should be prepared beforehand in every clinic and hospital should not miss such cases and treat them properly when diagnosed.

Due to the limited supply of vaccines, vigorous outbreak management and other prevention strategies should be prioritized at first. This should include vaccination among higher-risk cohorts such as men having sex with men, bisexuals, those who are in close physical contact such as intimate partners and housemates, medical and paramedical staffs. Ring vaccination as recommended by previous studies is one of the key strategies to contain the Monkeypox outbreak^24^. Moreover, the vaccine hesitancy among the general population of developed countries is high, which needs to be addressed by taking an example of COVID-19 pandemic where vaccination is the number one strategy to tackle the pandemic. Besides immunization, conducting awareness programs for safe sexual practices among homosexual partners is also essential to halt the monkeypox outbreak. Various articles have stressed on public education about possible risk factors and MPX prevention strategies in order to eliminate as a disease of public health concern^25,26^. Furthermore, it is necessary to implement a one-health approach by quickly assessing the risk associated with reverse zoonosis and Monkeypox transmission^27^.

Previously, The Centers for Disease Control and Prevention (CDC) announced that people with known heart disease should not take smallpox vaccines until further investigation. This announcement was based on reports of seven civilian vaccines suffering heart problems, like heart attack, angina, and myo/pericarditis^28^. It is not known whether similar conditions should be applied in case of MPX as well. Furthermore, in a previous pandemic due to COVID-19, mRNA COVID-19 vaccines were seen to be associated with myo/pericarditis. ATAGI recommends that the MPX vaccine (ACAM2000) and mRNA COVID-19 vaccine dose should be considered separated by several weeks. Still, with this latest outbreak, much more information is lacking. Despite being a mostly self-limited disease that can be prevented through immunization, various cardiovascular sequelae are emerging as a cause of concern relating to severe disease as well as monkeypox vaccines. It is imperative to increase investment and funding in research investigating the cardiovascular risk profile of the monkeypox vaccine as well as determine the factors responsible for the increased risk of cardiovascular sequelae after immunization with monkeypox. As MPX cases rise across the world, vaccines gain a prominent role and the cardiovascular concerns associated with the vaccine warrant further investigation which can ultimately guide early detection, management of cardiovascular events as well as enable a more organized vaccination process.

## Ethical approval

Not required.

## Consent

Not required.

## Source of funding

None.

## Author contribution

A.B.S. and A.M. conceptualized the topic, coordinated reading, writing and editing. All authors contributed to reading, writing, editing the original draft and critical revision. All authors contributed to various aspects of reading, data collection, writing the original draft and implementing changes for critical revision under the supervision of Abhigan Babu Shrestha and Aashna Mehta.

## Conflicts of interest disclosure

None.

## Research registration unique identifying number (UIN)

Not required.

## Guarantor

Pashupati Pokharel.

## Provenance and peer review

Not commissioned, externally peer-reviewed.

## Data availability

All data are presented within the manuscript.
